# Supplementary material for: Absolute quantitation of individual SARS-CoV-2 RNA molecules provides a new paradigm for infection dynamics and variant differences
Source: eLife. 2022 Jan 20;11:e74153. doi: 10.7554/eLife.74153 (PMC8776252; doi:10.7554/eLife.74153)
Supplement: Supplementary file 1. [file elife-74153-supp1.pdf]

Supplementary Data Item

Multiple sequence alignment of SARS-CoV-2 variants of concern and variants of interest centred around transcriptional regulatory sequence motifs.

Occurrence of 10nt motif “CTAAACGAAC” in SARS-CoC-2 in relation to transcriptional skipping, ORF annotations, and mutations in different SARS-CoV-2 variants.

Top panels: Position of transcriptional skip ends (landing site) and their usage as percent of all skip events in B.1.1.7 (blue) and Victoria (orange) strains based on RNA-seq.

Middle panels: Multiple sequence alignment of SARS-CoV-2 reference strain, Victoria, and B.1.1.7 strains, as well as other variants of concern or variants of interest. 10nt motif “CTAAACGAAC” and its near variants are indicated by light blue boxes. Annotated ORF start and end codons are indicated in green and gray, respectively. Mutations relative to the reference strain are shown in yellow boxes. Bases with at least 0.1% skip site usage in B.1.1.7 (blue) or Victoria (orange) are indicated.

Bottom panels: Number of variants with mismatches relative to the reference strains at any given position.

66 – 75 motif

Mutations, motifs, annotations, and skip site usage in Victoria and B.1.1.7

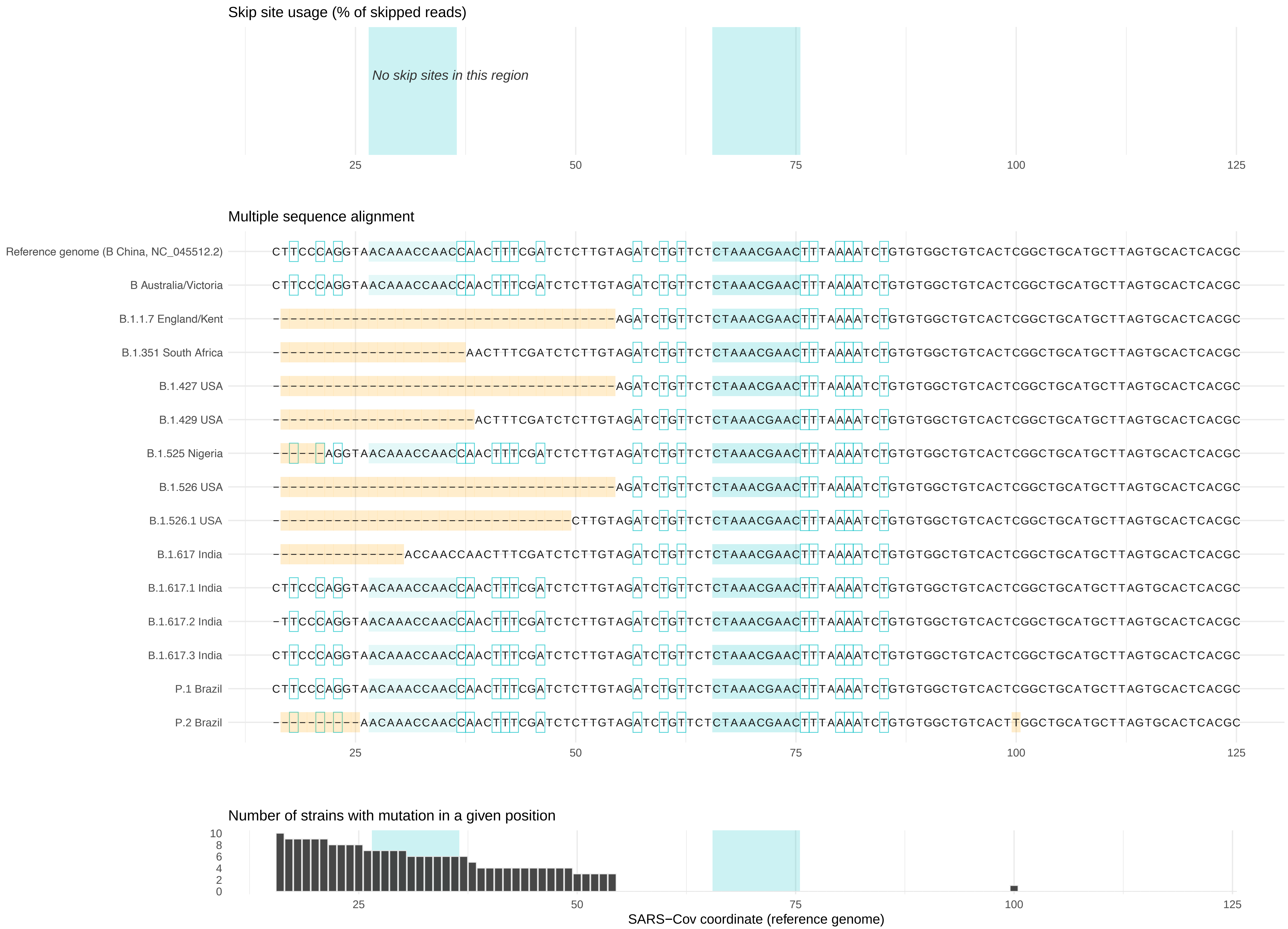

Highlighted regions indicate mutations

Annotations are based on reference genome

21552 – 21561 motif

Mutations, motifs, annotations, and skip site usage in Victoria and B.1.1.7

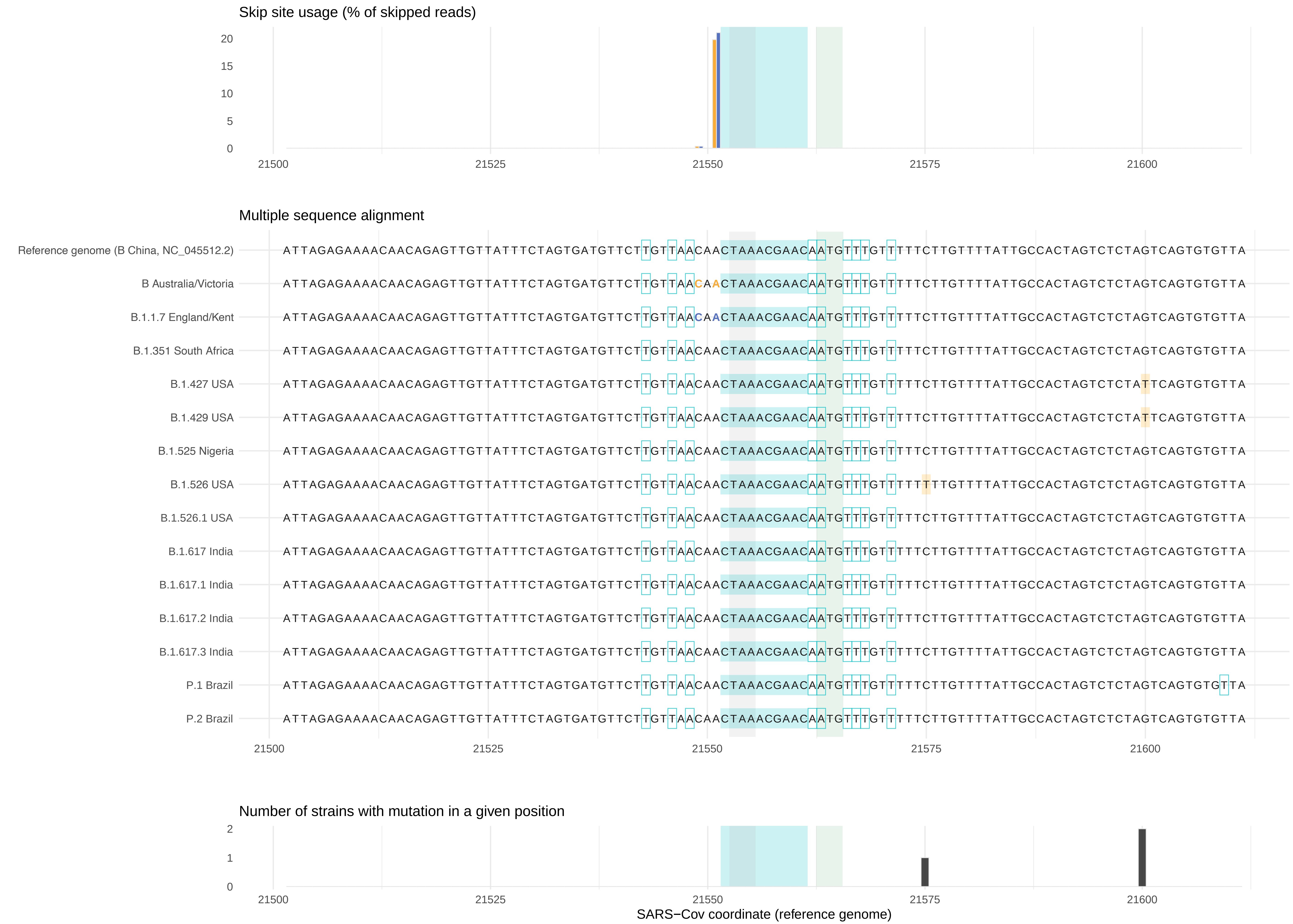

Highlighted regions indicate **motifs**, **start codon** (S), **stop codon** (ORF1ab), **mutations**

Annotations are based on reference genome

25381 – 25390 motif

Mutations, motifs, annotations, and skip site usage in Victoria and B.1.1.7

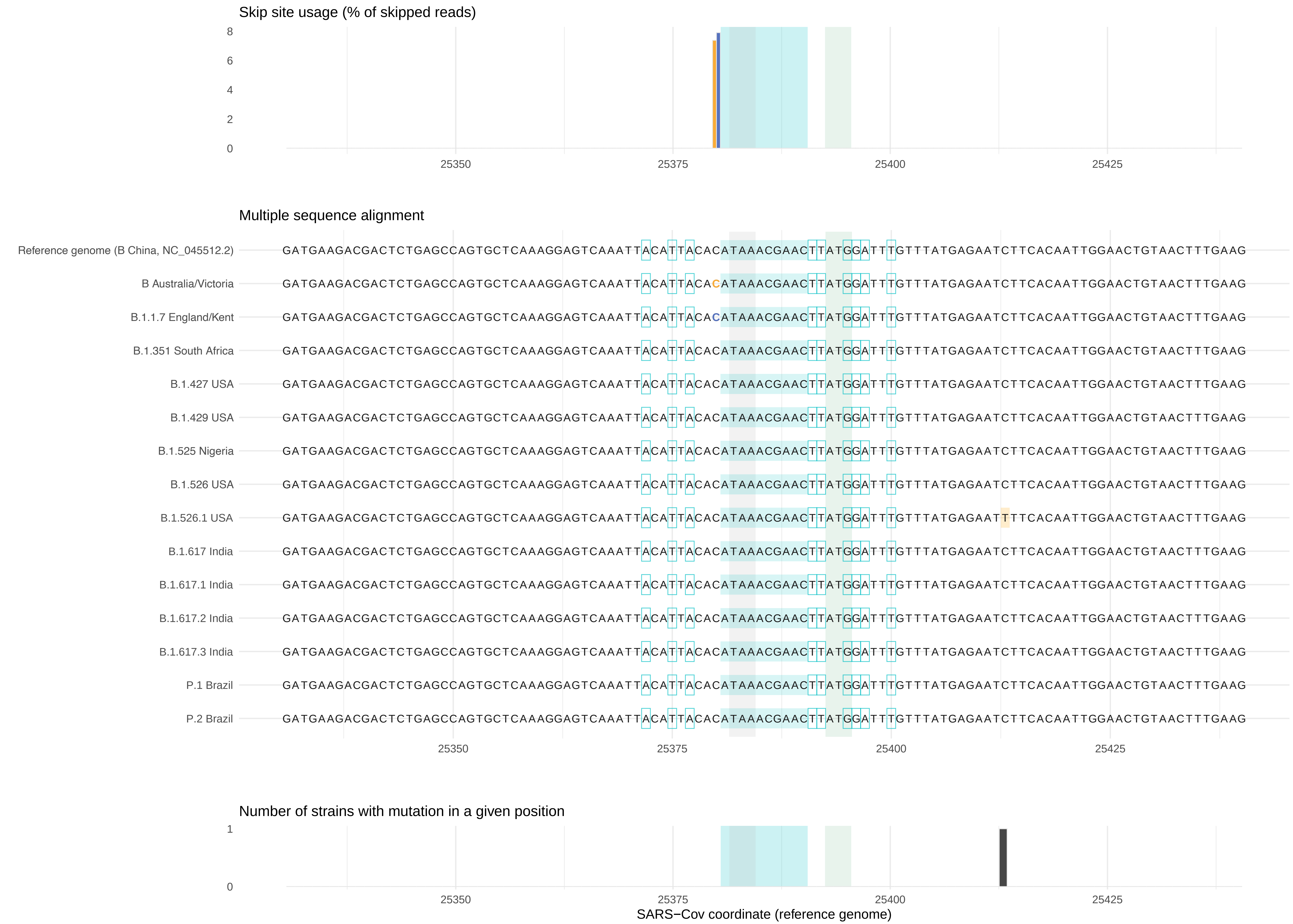

Highlighted regions indicate **motifs**, **start codon** (ORF3a), **stop codon** (S), **mutations**

Annotations are based on reference genome

26469 – 26478 motif

Mutations, motifs, annotations, and skip site usage in **Victoria** and **B.1.1.7**

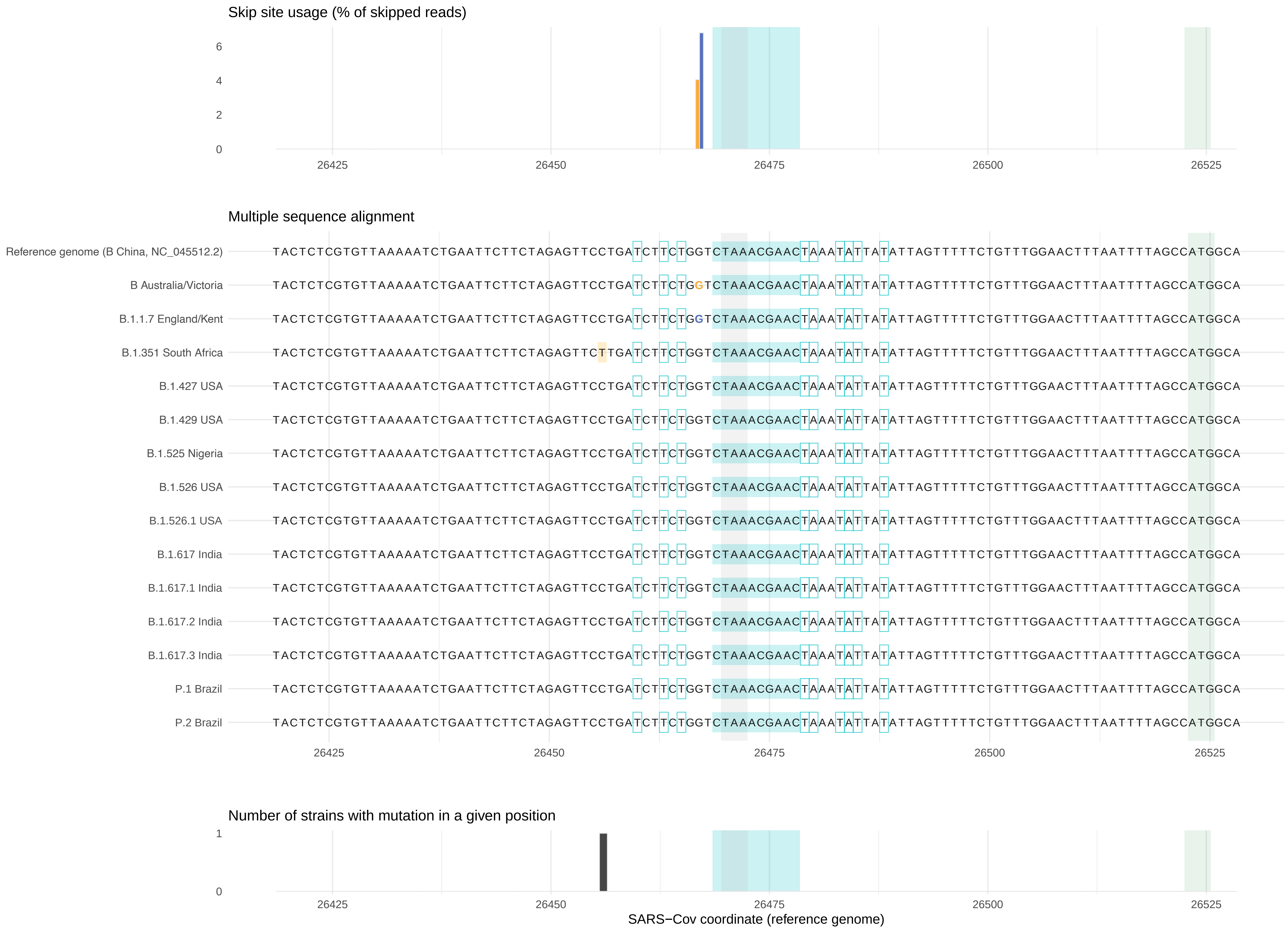

Highlighted regions indicate **motifs**, **start codon** (M), **stop codon** (E), **mutations**

Annotations are based on reference genome

27384 – 27393 motif

Mutations, motifs, annotations, and skip site usage in Victoria and B.1.1.7

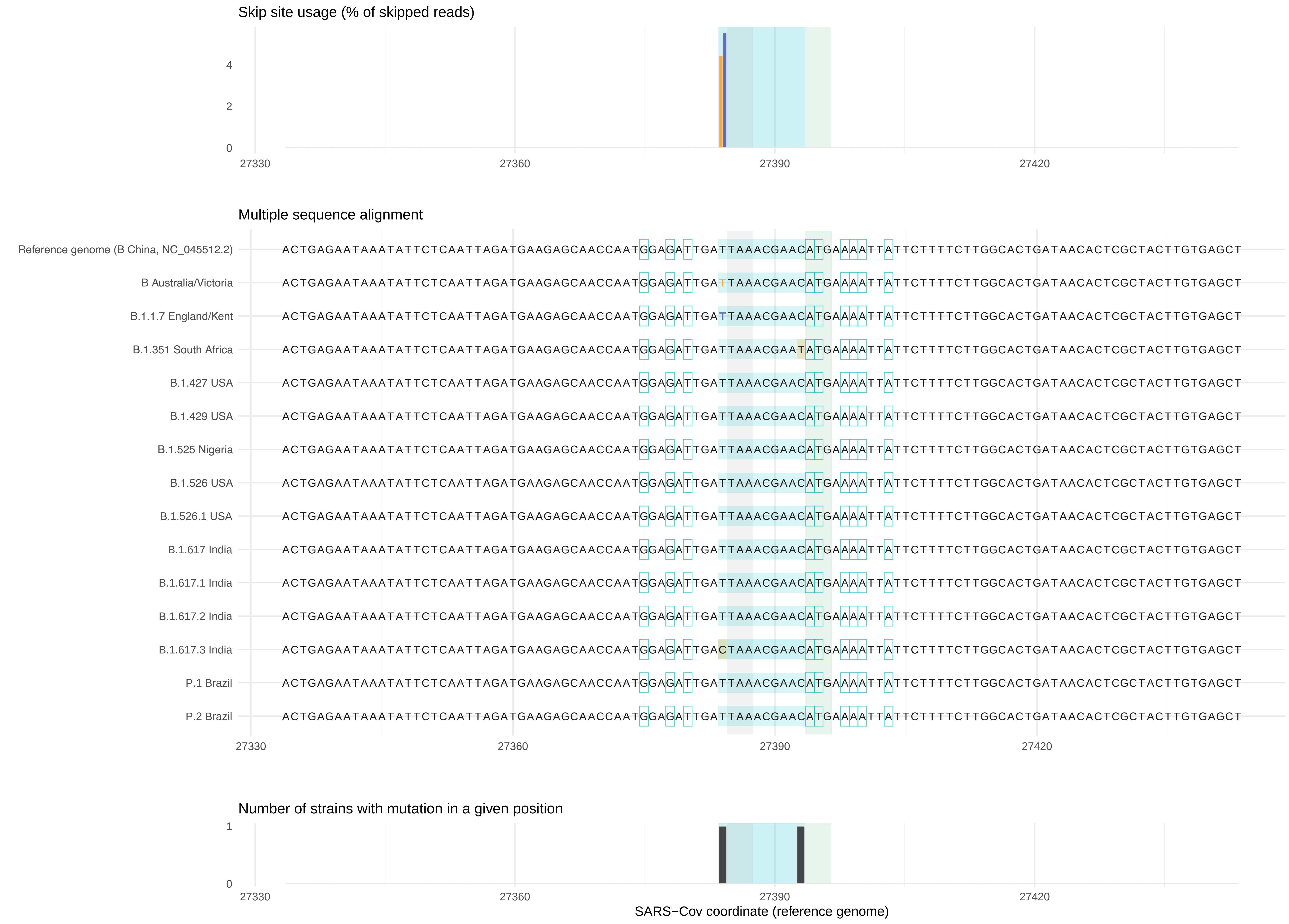

Highlighted regions indicate **motifs**, **start codon** (ORF7a), **stop codon** (ORF6), **mutations**
Annotations are based on reference genome

27884 – 27893 motif

Mutations, motifs, annotations, and skip site usage in Victoria and B.1.1.7

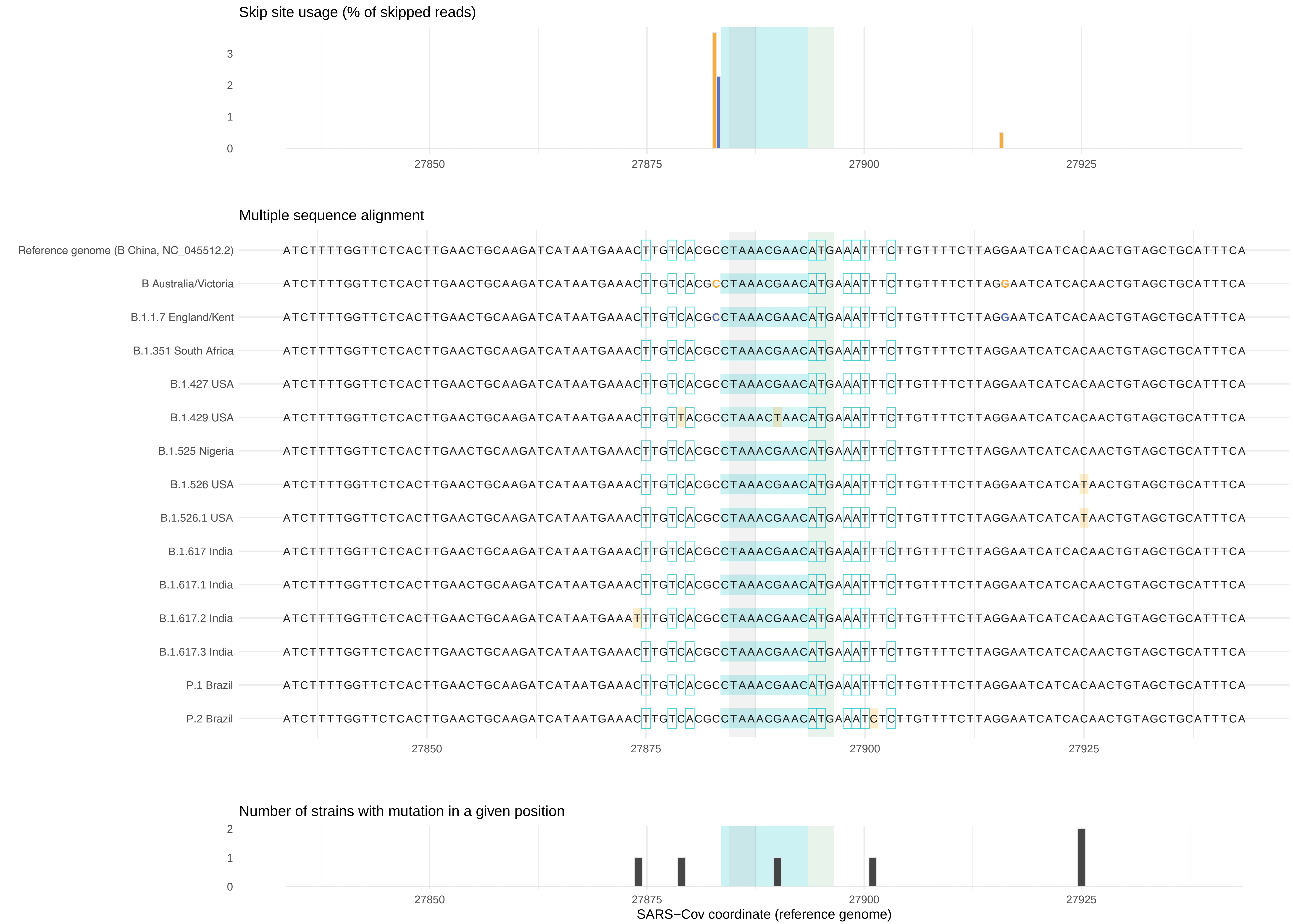

Highlighted regions indicate **motifs**, **start codon** (ORF8), **stop codon** (ORF7b), **mutations**

Annotations are based on reference genome

28256 – 28265 motif

Mutations, motifs, annotations, and skip site usage in Victoria and B.1.1.7

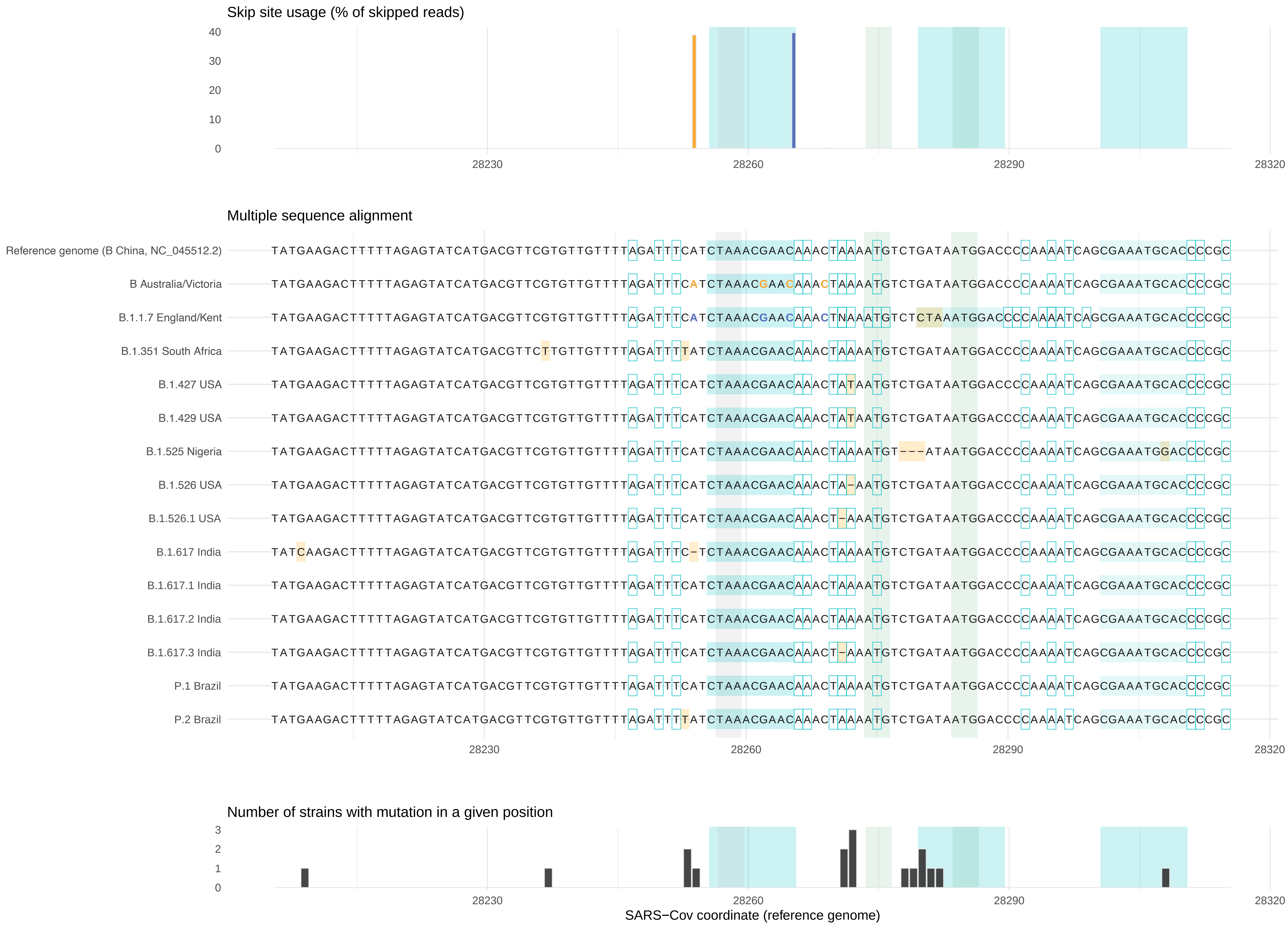

Highlighted regions indicate motifs, start codon (N, ORF9b), stop codon (ORF8), mutations

Annotations are based on reference genome

28878 – 28887 motif

Mutations, motifs, annotations, and skip site usage in Victoria and B.1.1.7

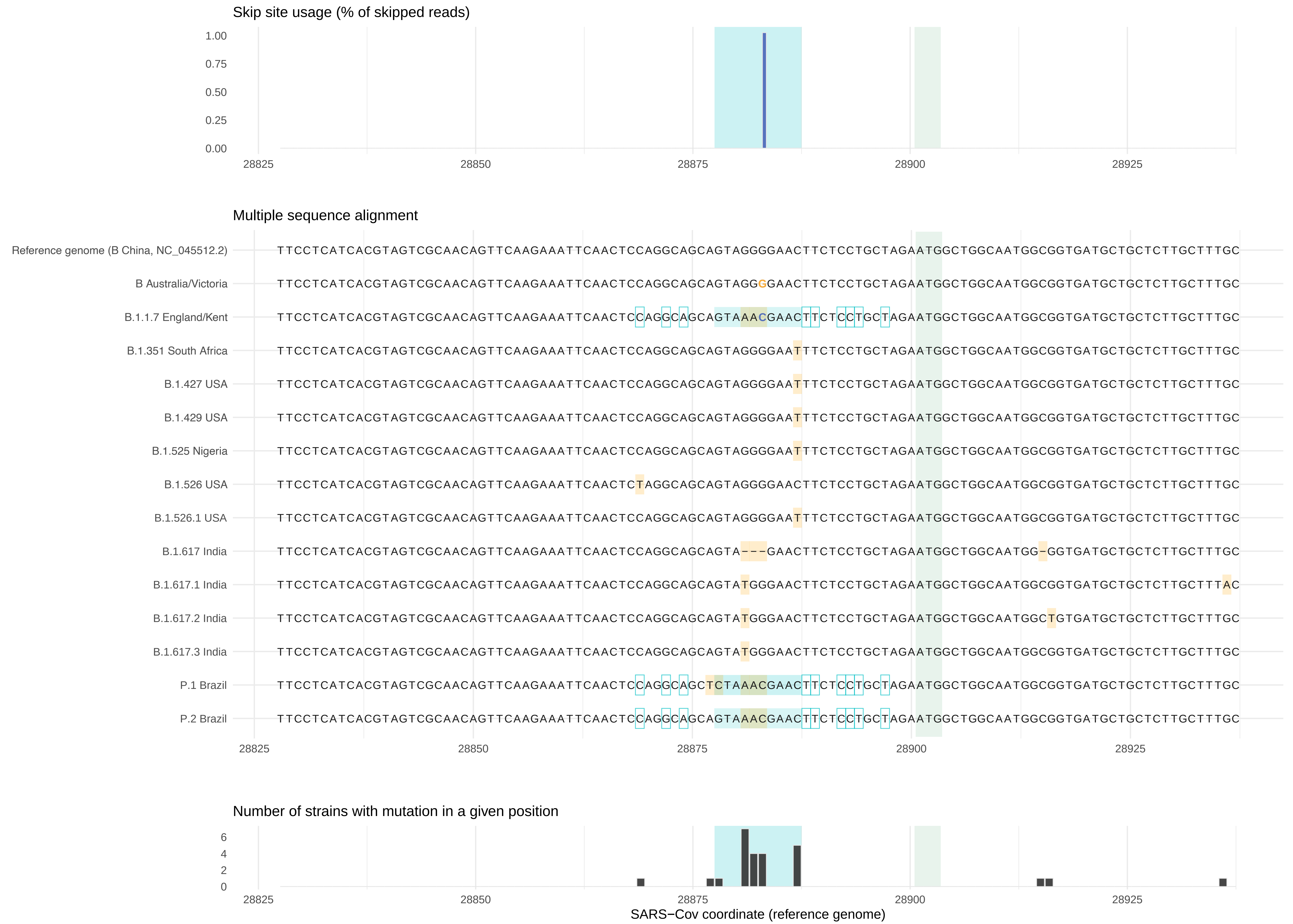

Highlighted regions indicate motifs, start codon (N*), mutations

Annotations are based on reference genome
